# Local Lidocaine 2% in Postoperative Pain Management in Cesarean Delivery

**Published:** 2015-03

**Authors:** Mandana Mansour Ghenaee, Shaghayegh Rahmani, Mina Jafarabadi

**Affiliations:** 1 Guillan University of Medical Sciences, Rasht, Iran; 2 Patient Safety Research Center, Mashhad University of Medical Sciences, Mashhad, Iran; 3 Tehran University of Medical Sciences, Tehran, Iran

**Keywords:** lidocaine 2%, cesarean section, VAS

## Abstract

**Objective:** To evaluate the ability of lidocaine local injection to relieve postoperative pain in cesarean delivery.

**Materials and methods:** This double blinded clinical trial was conducted in a university on 100 women underwent elective cesarean section during March 2012 till March 2013. Patients were divided to two groups with random block method (n = 50 in each group). First group received 4mg/kg lidocaine 2% and its volume was titrated to 30 cc with distilled water. Second group received 30 cc of normal saline. Injections were done in different layers of abdominal wall. Visual analogue scale (VAS) was applied to record 12 hours severity of pain in all patients. Data were analyzed by SPSS software using one way ANOVA parametric test.

**Results:** VAS average was 4/13 in lidocaine group and 4/81 in placebo group. The need for analgesic use was 300 mg in lidocaine and 346 mg in normal saline patients and the difference was significant (p < 0.000).

**Conclusion:** Local use of lidocaine 2% in cesarean incision reduced post operative pain and need to use analgesic agents.

## Introduction

Cesarean section is the most common major surgery which is performed for women in the United States currently, and it is estimated that more than 1.3 million cesareans performed annually. Postoperative pain relief is an important consideration issue as in clinic. Although different methods have been described for proper pain relief, it is not sufficient and satisfactory in some patients (1, 2). Due to several aspects such as maternal and neonatal wellbeing, postoperative pain relief in cesarean delivery is crucial. Providing a proper and efficient pain management is necessary during hospitalization which prevents cesarean section related complications which could affect breastfeeding and mother and neonate health status (3). 

Several studies have been conducted to evaluate the efficacy of different post-partum pain management protocols for cesarean section (4). On the other hand, pain control method depends on individual variability, such as age, genetic and psychological factors and also sensitivity to pain. These methods might vary in different region and center regard to their facilities (2, 3). 

Local analgesics usage during surgery has fewer side effects in compare with opioids or neuro-axial method (1). Considering the pain control after cesarean section and drug availability and pharmacologic evidence, we conducted the present study to examine the effectiveness of 2% lidocaine infiltrated in incision site for pain control in cesarean section postoperative.

## Materials and methods

After ethics committee approval, we randomly (random block method) assigned 100 consecutive patients undergoing elective cesarean section to one of the two equal groups. Surgeon and nurse who evaluated pain were blinded about the injected solution. 

Primigravid women, with the weight between 50 and 80 kg, and minimum education level about high school were selected and patients with comorbidity, contraindication for spinal anesthesia and drug abuse were excluded and also surgeries which lasted more than one hour. 

Group A: First group received 4mg/kg from lidocaine 2% (solution volume was titrated to 30 cc with distilled water) and it was divided into three parts and the first two parts were injected in abdominal muscle and subcutaneous layer. Third part was infiltrated on peritoneum.

Group B: second group received 30 cc of normal saline, and then this was injected in different layer of abdominal wall. Third part of it was infiltrated on peritoneum. 

Visual Analog Scale for Pain was described for all patients before operation and they were asked to estimate their post operative pain between zero and 10. VAS was recorded for 12 hours after surgery as below: every one hour in the first 4 hour after surgery, and every 4 hour for the rest of the time. If the patient complained from pain, a 100 mg diclofenac suppository was administered and time was recorded. 

Cesarean section technique: All operations were performed by a single surgeon. The surgery technique and postoperative protocol were same. Cesarean section was performed with transverse lower segment incision and anesthesia method was spinal in all cases. 

Statistical analysis: comparison between groups was performed using student’s t-test or one way ANOVA parametric test. Descriptive analysis were computed for all variables. P value less than 0.05 was considered significant statistically.

## Results

Demographic variables were matched in two groups. Patients mean age was 24 ± 4 years and their weight was 61 ± 4 kg. Mean duration for removing urinary catheters and walking after surgery was 18 ± 2 hours in both groups. There was no adverse event or side effect in patients. Mean VAS had no difference between groups at the first hour which might result from remaining effect of spinal anesthesia. 

Mean VAS was 4.13 and 4.81 in lidocaine and normal saline patients, respectively. VAS in lidocaine group was significantly higher at the second, third, fourth and overall 12 hour score (p < 0.000). Mean suppository usage varied between groups (p < 0.00). Mean dosage of analgesic needed for pain relief after CS was 300 mg in lidocaine group and 346 mg in normal saline (p < 0.00). 

Mean pain score of VAS had a normal distribution in both groups which is compared between groups in a 12 hours period in table 1.

**Table 1:** Mean VAS was compared between groups in a 12 hours period

**Time**	Mean VAS	SD	**F**	**p Value**
First hour	**3.56**	**2.51**	0.811	0.448
	**4.06**	**2.75**		
Second hour	**4.16**	**1.26**	16. 07	0.000
	**5.3**	**1.2**		
Third hour	**4.8**	**1.21**	19.91	0.000
	**5.6**	**1.82**		
Forth hour	**4.4**	**1.59**	17.41	0.000
	**5.4**	**1.61**		
Fifth hour	**4.2**	**1.22**	0.515	0.599
	**4.4**	**1.25**		
Twelfth hour	**3.6**	**1.1**	2.04	0.136
	**3.9**	**0.99**		
Overall	**4.1**	**0.68**	23.82	0.000
	**4.1**	**0.71**		

**Table 2:** The mean interval between the completions of the operation and need to use diclofenac suppository

**Group**	**Mean (hour)**	**SD**	**p value**	**F**
Lidocaine	2.53	1.4	0.016	4.36
Normal saline	1.53	0.68		

VAS in lidocaine group was significantly higher at the second, third, fourth and overall 12 hour score (p < 0.00). Mean suppository usage varied between groups (p < 0.00). 

The mean interval between the completion of the operation and need to use diclofenac suppository between two groups are compared in table 2.

The mean interval between the completions of the operation and need to use diclofenac suppository was significantly less in lidocaine group (p = 0.016). Turkey post HOC test confirmed that diclofenac suppository usage at first hour showed no difference in groups.

## Discussion

This study showed that Lidocaine 2% injection in cesarean section incision results in reduction in post operative pain and analgesic usage without any important side effect. 

Different studies approved our results. Moradi and colleagues (5) showed that lidocaine could not relief dental root pain as effective as marcaine in first 36 hour. Lowenstein confirmed that lidocaine could reduce pain after hysterectomy, particularly in the first 8 hour after surgery (6). Rosaeg showed that marcaine had better effect in post operative pain control in cesarean section patients at 4, 6 and 9^th^ hours and opioids usage was lesser in marcaine group in regard to placebo (7). Beaussier also proved that marcaine infiltration during abdominal surgeries could lessen analgesic need after operation (8). 

There are few evidence which are opposed the idea about the efficacy of local analgesic in post operative pain control. In a study by Abbas et al extra peritoneal bupivacaine usage in laparoscopic herniorrhaphy did not lead to reduction in analgesic dosage and pain (9). This difference might happen due to our methodology in which we used VAS and he used SF-MPQ. 

Post operative pain control has a great impact on patient satisfactory and well being which has a great impact on the quality of maternal and neonatal care. 

## Conclusion

Lidocaine 2% injection in cesarean section incision results in reduction in post operative pain and analgesic usage without any important side effect. 

## References

[1] Cunningham FG, Kenneth J (2010). Williams Obstetrics.

[2] Ronald D, Miller RD (2758-71). Miller's anesthesia.

[3] Ritter J, Lewis L, Mant T, Ferro A (2008). A Textbook of Clinical Pharmacology and Therapeutics.

[4] Gibbs R, Karlan B, Haney A, Nygaard I (2003). Danforth's Obstetrics & Gynecology.

[5] Moradi S, Naghavi N (2010). Comparison of bupivacaine and lidocaine use for postoperative pain control in endodontics. Iran Endod J.

[6] Lowenstein L, Zimmer EZ, Deutsch M, Paz Y, Yaniv D, Jakobi P (2008). Preoperative analgesia with local lidocaine infiltration for abdominal hysterectomy pain management. Eur J Obstet Gynecol Reprod Biol.

[7] Rosaeg OP, Morrison S, MacLeod JP (1996). Anaesthetic management of labour and delivery in the parturient with mitochondrial myopathy. Can J Anaesth.

[8] Beaussier M, Bouaziz H, Aubrun F, Belbachir A, Binhas M, Bloc S (2012). [Wound infiltration with local anesthetics for postoperative analgesia. Results of a national survey about its practice in France]. Ann Fr Anesth Reanim.

[9] Abbas MH, Hamade A, Choudhry MN, Hamza N, Nadeem R, Ammori BJ (2010). Infiltration of wounds and extraperitoneal space with local anesthetic in patients undergoing laparoscopic totally extraperitoneal repair of unilateral inguinal hernias: a randomized double-blind placebo-controlled trial. Scand J Surg.

